# Might Starvation-Induced Adaptations in Muscle Mass, Muscle Morphology and Muscle Function Contribute to the Increased Urge for Movement and to Spontaneous Physical Activity in Anorexia Nervosa?

**DOI:** 10.3390/nu12072060

**Published:** 2020-07-10

**Authors:** Regina C. Casper

**Affiliations:** Department of Psychiatry and Behavioral Sciences, Stanford University School of Medicine, Stanford, CA 94305, USA; rcasper@stanford.edu

**Keywords:** anorexia nervosa, human undernutrition, skeletal muscle, muscle morphology and function, work capacity, urge for movement, restlessness, physical activity, drive for activity, experimental semi-starvation

## Abstract

Severely undernourished and underweight anorexia nervosa (AN) patients typically remain active and mobile. Might such persistent physical activity in AN be supported by specific adaptations in muscle tissue during long term undernutrition? To identify potential differences, studies examining the effects of undernutrition on skeletal muscle mass, muscle morphology and muscle function in healthy humans and in AN patients were reviewed. Adjustments in muscle morphology and function in AN did not differ in substance from those in healthy humans, undernourished people, or undergoing semi-starvation. Loss of muscle mass, changes in muscle contractility and atrophy of muscle fibers (predominantly type II fibers) characterized both groups. Muscle innervation was unaffected. Work capacity in men in semi-starvation experiments and in females with AN declined by about 70% and 50%, respectively. Perceptions of fatigue and effort distinguished the groups: signs of general weakness, tiring quickly and avoidance of physical activity that were recorded in semi-starvation were not reported for AN patients. The absence of distinctive starvation-related adjustments in skeletal muscle in AN suggests that new methods, such as muscle gene expression profiles in response to deficient nutrient intake, and better knowledge of the central regulatory circuitries contributing to motor urgency will be required to shed light on the persistent mobility in AN patients.

## 1. Introduction

Anorexia nervosa (AN), a disorder principally of female adolescents, is characterized by prolonged self-imposed restriction of food intake leading to significant weight loss and an emaciated appearance, the result not only of loss of fat tissue but also of muscle tissue. The body responds to the caloric restriction with adaptive metabolic and physiological responses not different from those found in starvation [1]. Many adjustments, such as hypothermia, hypotension, bradycardia, and a low resting metabolic rate, are aimed at preserving energy [2]. Despite high rates of dieting among teenagers, the incidence of AN remains low [3]. The apparent ease with which previously healthy teenagers lose up to 40% of their body weight poses the question whether other factors besides a restricted caloric intake might facilitate weight loss in AN.

A recent genetic study [4] nested AN within the correlational structure of compulsive psychiatric disorders, pointing to motion as a marker. Actually, the earliest clinical descriptions refer to the persistence of movement in cachectic AN patients. In Lasègue’s words: “another ascertained fact is, that so far from muscular power being diminished, this abstinence tends to increase the aptitude for movement” [5]. Janet [6] writes: “It is the exaggerated need for physical movement which accompanies true anorexia nervosa”. The contemporary fitness culture tends to explain high activity levels in emaciated AN patients as a willful strategy to increase caloric expenditure. From a physiological point of view, however, hyperactivity [7], excessive exercise [8], and even spontaneous daily activity levels not different from normal [9] under extreme fasting conditions appear incompatible with “the muscular power being diminished”. The increased urge for movement and restlessness [10,11] and normal spontaneous physical activity levels in emaciated AN patients appear to be generated by neurophysiological and/or metabolic changes during starvation and suggest an anomaly in the biobehavioral regulation of the adaptation to starvation in AN [12]. The underlying mechanisms are not known, except for the contribution of low leptin plasma levels [13]. To our knowledge, the hypothesis that specific starvation-induced adaptations in the skeletal musculature of AN patients might create conditions that promote restlessness and persistent mobility has not been examined.

## 2. Aims of the Review

The present review will describe and compare studies in healthy populations exposed to undernutrition with studies in AN patients in order to identify potential differences in starvation-induced changes in skeletal muscle tissue, muscle morphology and function which may provide direction to investigate the increased urge for movement and the spontaneous activity in AN.

Publications on undernutrition in healthy humans will be summarized first, followed by studies in AN.

## 3. Results

### 3.1. A Brief Synopsis of the Metabolic Adaptations in Prolonged Semi-Starvation/Starvation

Under fasting conditions, following significant restriction of food intake, a low blood sugar level leads to decreased secretion of insulin and increased secretion of glucagon [14]. With glucose levels insufficient to provide glucose to the brain and other tissues, after several weeks of starvation, ketone bodies become the major fuel for the brain. The dominant metabolic processes are the mobilization of triacylglycerols in adipose tissue and gluconeogenesis by the liver. The liver obtains energy for its own needs by oxidizing fatty acids released from adipose tissue. The concentrations of acetyl CoA and citrate consequently increase, which switches off glycolysis [15]. After about three days of starvation, the liver forms large amounts of acetoacetate and beta-hydroxybutyrate and releases large amounts of these ketone bodies into the blood. The brain also begins to consume appreciable amounts of acetoacetate in place of glucose. After several weeks of starvation, ketone bodies become the major fuel for the brain.

The uptake of glucose by muscle is markedly diminished because of the low insulin level, whereas fatty acids enter freely. Eventually, muscle shifts almost entirely from glucose to fatty acids for fuel. Short term fasting is associated with an increased release of glucogenic amino acids from muscle [16]. Measurements of amino acid balance across the forearm muscle have shown an adaptive reduction in muscle amino acid release with a longer duration of caloric restriction [17]. Muscle loss must be minimized, since survival for most animals and mammals depends on being able to move. Eventually, the heart also uses ketone bodies as fuel. The more effective the conversion of fatty acids into ketone bodies by the liver, the less muscle is degraded. The critical component of loss in skeletal muscle in humans is its link to impaired functional performance and work capacity.

### 3.2. Body Weight and Muscle Mass after Prolonged Starvation

Marked atrophy of the skeletal musculature is a prominent sign of severe undernutrition [18] and weight loss in excess of 25% is regularly associated with significant loss of muscle mass [19]. Two experiments in healthy humans exposed to controlled prolonged food restriction provide information about changes in skeletal muscle (both muscle function and physical activity); both were conducted in young adult men.

#### The Carnegie and Minnesota Semi-Starvation Experiments

The Carnegie experiments [20] were directed toward proving successful adaptation of the organism to a restricted diet and were centered on the basal metabolic rate. Under ordinary conditions, the energy needs for a 24 h period for a healthy well-nourished 70 kg man range from about 1600 kcal to 6000 kcal. The Carnegie experiments aimed at a 10% weight loss with gradual caloric reduction initially at 2200 kcal, ultimately lowered to 1500 kcal over the three winter months of 1917/18. Diet and exercise were monitored by the participants, who signed an honor code. Twelve college students (Squad A) ultimately achieved a 12% weight loss from their initial body weight of 67 kg. Calculations estimated the loss of muscle mass to be half of the maximum weight loss of 8.2 kg, based on measurements of circumferences of the limbs and the trunk. Throughout the weight loss period, low amounts of nitrogen were lost in every subject. Physiologically, even in the absence of a significant decrease in body temperature, participants had an extreme sensitivity to cold; the basal metabolism declined by about 15%. At the lowest weight, physical endurance measured as grip strength was reduced by 8%.

The Minnesota semi-starvation experiment [21] was designed to reproduce famine during wartime in order to study not only the physical, but also the emotional and moral consequences of food deprivation, and is by far the most carefully conducted and best documented study. Thirty-six men were recruited among conscientious objectors to undergo a three-month control period followed by a six-month semi-starvation phase to bring about a 25% weight loss of their ideal body weight, then followed by three months of restricted rehabilitation. Unlike the Carnegie experiments, nutrient and caloric intake were strictly monitored and supervised by the researchers with prescribed, albeit not supervised, daily physical activity. Starting from an average energy intake of 3500 kcal/day at an average initial weight of 69 kg, the men lost on average 16 kg within the six months of semi-starvation on an average intake of 1500 kcal/day. Muscle mass loss estimated from measurements of the mid upper arm and thigh corrected for bone was 41% of the control value and fat loss 69% of the control value based on the specific gravity of the body. Muscle mass of the upper extremity tended to atrophy somewhat more rapidly than the lower extremity or the total body muscle mass. Cumulative nitrogen loss was calculated as an average daily loss of 3.8 g (total 1990 g) during the first 12 weeks and 1.3 g (total 690 g) for the second 12 weeks of semi-starvation. Work capacity and running time as measured by the Harvard Fitness test declined to 28% and 21% of the control value, respectively. Calculations arrived at a 70% reduction in the calories available for work.

### 3.3. Skeletal Muscle Function and Histological Features in Humans on Hypocaloric Diets and after Re-Nutrition

#### 3.3.1. Electrophysiological Testing of Muscle Function in Undernutrition in Healthy Subjects, Malnourished Patients and Obese Individuals

Lennmarken et al. [22] investigated whether changes in skeletal muscle function could be used to assess malnutrition in healthy individuals. Eleven women, at a mean age of 34 years and a mean BMI (body mass index in kg/m^2^) of 23.6, underwent five days of total starvation while continuing their work as lab personnel. At a mean weight loss of 3.3 kg, hand grip strength was significantly reduced. On electrical stimulation of the ulnar nerve, a higher contraction force at low (10 Hz) frequency stimulation, a longer relaxation rate and reduced endurance in the adductor pollicis muscle were found. Since the changes were small, the authors concluded that muscle function tests were not sensitive enough to diagnose short term undernutrition.

Lopes et al. [23] used the same methodology in 10 adult malnourished patients with gastrointestinal disorders and 22 age-and sex-matched healthy control subjects. Again, at the lowest (10 Hz) stimulation, malnourished patients showed a significant increase in the force of contraction, but no differences were found at higher stimulation frequencies in relation to controls. The muscle relaxation rate was slower and muscle fatiguability was markedly increased in patients compared to controls; muscle endurance testing showed significant force loss; all parameters improved in four patients who were re-studied after re-nutrition.

Function tests of the adductor pollicis muscle in six obese patients on a baseline diet, repeated after two weeks on a 400-kcal/day diet, followed by two weeks of total fasting and then two weeks of re-feeding, similarly showed increased force of contraction at 10 Hz during both fasting conditions compared to baseline [24]. The patients lost on average 6.5 kg and 11.5 kg, respectively, from an original weight of 120.5 kg. Muscle voluntary force was unchanged and maximal muscle force with electrical stimulation was reduced after total fasting, with greater muscle fatiguability. Re-feeding led to rapid normalization of all muscle function parameters.

By contrast, Newham et al. [25] studied twenty-one obese patients with an initial mean weight of 117.2 kg, maintained on a daily 450 kcal diet (containing 32 g protein) for two weeks, resulting in an average 5.7 kg weight loss. No significant changes in maximum voluntary contraction, nor in force:frequency characteristics after electrical stimulation of the adductor pollicis muscle, nor in fatigability in comparison to normal subjects, were found. Interestingly, a trend was noticeable for the force:frequency relationship at 10 Hz to increase over the 12 days of starvation. The authors indicate that dietary differences might account for the divergent findings in these two otherwise similar studies, because in three patients in Russel’s [24] study, the hypocaloric diet consisted entirely of protein and in the remaining three, entirely of carbohydrates.

#### 3.3.2. Histological, Histochemical and Biochemical Analyses of Muscle Structure in Human Undernutrition

Human skeletal muscle fibers have been classified through histochemical staining for pH-sensitive actomyosin ATPase activity into type I (slow twitch) and type II (fast twitch) fibers. Type II has been broadly categorized into type IIA, type IIB and type IIC, but other subclasses also exist. Type I muscle fibers tend to be oxidative, insulin-sensitive and vascularized, whereas type II B fibers are glycolytic.

Light and electron microscopy showed that in infants and children with protein-caloric malnutrition, a reduction in fiber size in the sartorius muscle was seen, which Dastur et al. [26] attributed to a slowing down in development for a given age. Nassar et al. [27] observed in marasmic infants, in addition to fiber atrophy, fragmentation and degeneration of myofibrils in the sartorius muscle. In both studies, the children suffered from other ill-defined nutritional deficiencies.

In healthy adults, Russell et al. [28] biopsied the gastrocnemius muscle in five out of six obese female subjects maintained on a 400 kcal hypocaloric diet for two weeks. Histochemical examination revealed significant fiber atrophy, predominantly of type II fibers, compared to baseline values. No significant changes in muscle glycogen, lactate, pyruvate, or free energy stores were observed. Muscle enzymes, however, were significantly decreased and there was loss of muscle force. Intracellular muscle calcium was significantly increased, leading the authors to speculate that altered calcium kinetics might have contributed to slower muscle relaxation rates and greater fatigue.

### 3.4. Physical Fitness and General Well-Being in Healthy Humans during Semi-Starvation

In the Carnegie Experiments [20] in the absence of a significant decrease in body temperature, participants experienced an extreme sensitivity to cold, requiring extra clothing; basal metabolism declined by about 15%. At the lowest weight, physical endurance during daily activities was slightly affected. Subjectively, the men complained about general weakness, of weakness of the legs, particularly in climbing stairs, lack of “pep” and fatigue.

In the Minnesota experiments, Keys et al. [21] used a complaint inventory to systematically record subjective signs in regularly scheduled interviews. At their lowest weight, nearly all men reported unsteadiness in walking, tiring quickly, general weakness, muscle soreness, not being able to work as before, avoidance of exercise, and a reduction in the “activity drive”. Moreover, personal appearance and grooming deteriorated and the men became indecisive, reluctant to plan and participate in social activities. Unsteadiness of footing in walking and inability to do hard physical work were universal at the end of the starvation period. Psychologically, a highly significant rise in the hypochondriasis, depression and hysteria scores on the Minnesota Multiphasic Personality Inventory (MMPI) was observed. The scores returned to normal values with re-nutrition and weight gain. When the men were interviewed years later, no psychiatric disorders were recorded [29].

### 3.5. Anorexia Nervosa (AN): Energy, Macronutrient and Micronutrient Intake

Daily energy needs for a healthy well-nourished female teenager range from 1800 kcal (7560 kJ) to 2400 Kcal (10,080 kJ) depending on the extent of her physical activity.

It is generally accepted that the reduced food intake in AN is not a result of loss of appetite; in fact ‘anorexia’ is not a diagnostic symptom of AN. Hunger feelings fluctuate in intensity and AN patients think and dream about food. Adolescents who ultimately develop AN eat increasingly less in order to lose weight, but rarely fast altogether. Males more often resort additionally to excessive exercise to lose weight [30].

Two factors ought to be mentioned that add variability in the reviewed studies: the duration of illness and the dieting subtypes of AN. AN typically has its onset during adolescence; studies in adults, therefore, indicate a chronic condition. Regarding the subtypes, nearly all studies recruited exclusively restricting AN patients, who tend to maintain a negative metabolic balance. Very few bulimic AN patients, whose periodic excessive intake creates a different nutritional milieu, were included. All AN patients in the studies were female, except for Essen et al. [31].

The common overriding factor for determining food preferences and avoidance of food in AN is the caloric content of food. Food choices cover a wider range. Depending on the individual’s imagination, preferences and food fads, one person might live exclusively for months on apricot juice, another might eat nothing but hard-boiled eggs and salad for weeks and months. During the 1960’s carbohydrates tended to be shunned [32], whereas more recently, fat avoidance has become more common [33]. Given the patient’s secrecy regarding any food intake, reliable data on the caloric and nutrient intake cannot be collected until the AN patient becomes hospitalized; even then, treatment expectations might interfere.

When AN patients were allowed to select their own food in hospital for the first 3–7 days, the average caloric intake was 1031 kcal/day. On average 48.5% of the calories were obtained from fat, 33.3% from carbohydrates and 18.2% from protein [34]. When eating for 24 h was assessed in a laboratory setting, restricting AN patients consumed 828 ± 210 kcal/day whereas healthy control subjects ate 2274 ± 564 kcal/day. Expressed as macronutrients, restricting AN patients (compared to healthy controls) ate less fat (13% vs. 31%), more carbohydrate (73% vs. 57%), but similar amounts of protein (14% vs. 12%) [33]. Moreiras-Varela et al. [35] used 48-h recall and a food frequency questionnaire to study dietary habits in AN outpatients and controls. Overall energy intake showed a wide range from 148 kcal/d in restricting AN patients to 2933 kcal/day in bulimic/vomiting AN patients; controls also reported a wide range from 1115–2838 kcal. AN patients ate less fat (58 g vs. 97 g), fewer carbohydrates (158 g vs. 205 g), and less protein (68 g vs. 97 g). In a study by Chiurazzi et al. [36], restricting AN patients reported a low intake of fat (23.6 g vs. 62 g) and carbohydrates (128 g vs. 220 g), yet a close to normal protein intake (51 g vs. 68 g); energy intake barely exceeded resting energy expenditure (906 kcal ± 224 and 896 kcal ± 157, respectively). Hadigan et al. [37] found that AN patients tended to overestimate their caloric intake by about 300 kcal/day as opposed to controls by comparing a one-month diet history to one-day observed food intake.

Critical micronutrient deficiencies occur more often with longer illness duration. Zinc (64%) and vitamin D (54%) deficiencies are common while other vitamin deficiencies are less frequent [38,39,40,41,42].

### 3.6. Body Composition and Muscle Mass in AN

Studies have applied different methods for estimating body composition from anthropometry, such as skinfold thickness or mid upper arm circumference (with calculations of lean body mass (LBM) based on the two skinfold method), densitometry (underwater weighing), bioelectrical impedance analysis (BIA), and dual-energy X-ray absorptiometry (DXA). Mattar et al. [43] have drawn attention to the fact that no study in AN has used the four-compartment (4-C) model for assessing body composition, which would allow researchers to measure variations in hydration levels and bone mineral content, because techniques which separate body composition into fat mass (FM) and fat-free mass (FFM) assume that body hydration level and bone mineral content are both stable. Total body water as a percent of body weight has been found to be higher in AN and total muscle water was increased in AN when compared to total muscle water after re-feeding [44]. Furthermore, variations in exercise are not taken into account for calculating FFM.

Loss of body weight in AN in all studies is associated with loss of muscle mass. In 13 adolescents with AN (age: 14.2 ± 1.8 years; mean BMI: 15.8), regional lean tissue was 11–20% and fat mass was 56–66% lower than in controls. Although total lean mass in the controls was closely related to total body bone mineral content, the correlation was significantly lower in AN, suggesting that structural changes resulted in a weaker feedback mechanism between bone and muscle [45].

Polito et al. [44] observed severe loss of muscle mass (39.1/BW %) in 20 outpatients (mean age: 24 years; mean BMI: 15.3) in comparison to the muscle mass of 10 healthy women (45.1/BW %) (age: 25 years; mean BMI: 20.6). At a body weight of 77% of controls, the muscle mass and the fat mass of AN patients was 70% and 50% that of the controls, respectively. Creatinine output was reduced to 60% of the normal value. In 88 day-hospital AN patients (mean age: 28 years; mean BMI: 16.6; body fat: 15.1%), average muscle mass was significantly lower (37.6/BW % vs. 41.2/BW %) than in 116 healthy women (aged 28 years; mean BMI: 21.7) [46].

Protein nutritional status, as assessed by total body nitrogen (TBN) through prompt gamma in vivo neutron activation analysis (NAA), was found to be 73% ± 10 of that predicted for age in 23 patients with AN (mean age: 15.5; mean BMI: 15.3). Total body nitrogen correlated most significantly with body weight [47].

### 3.7. Skeletal Muscle Function and Histological Features in AN

#### 3.7.1. Electrophysiological Testing of Muscle Function in AN

In eight chronically ill AN patients (mean age: 24 years; mean weight loss: 42%; mean BMI: 12.6), McLoughlin et al. [48] showed incomplete isometric maximal voluntary contraction of the dominant femoris muscle measured by dynamometer. Voluntary activation examined by percutaneous stimulation of the thigh at a frequency of 1 Hz was reduced to one-third of the predicted values for body weight. In the tests of contractility, the contractile force of the muscle at 10 and 20 Hertz was greater in AN patients with a slower relaxation rate than in 10 healthy staff members (mean age: 23.2; mean BMI: 22.4).

Melchiorri et al. [49] studied muscle fatigue during isometric contraction at 80% of maximal voluntary contraction (MVC) in eight chronic AN patients (mean age: 24.9; mean BMI: 15.5) compared to seven healthy women (mean age: 29; mean BMI: 20.0). There were no differences in maximal voluntary contraction or endurance. Baseline conduction velocity and rate of change were lower in AN than in controls. Mean power frequency (MNF) rate of change during contractions was found to be greater in AN than in controls.

#### 3.7.2. Histological, Histochemical and Biochemical Analyses of Muscle Structure in AN Patients

The studies of skeletal muscle morphology and muscle function in AN patients were based on the expectation that advanced stages of AN would reveal effects of pure malnutrition on muscle [50]. Muscle biopsies were generally taken from the vastus lateralis of the quadriceps femoris muscle.

Essen et al. [31] studied 10 AN patients, equally divided by sex (age: 15.3; BMI: 14.3). All muscle fiber types showed a marked degree of atrophy (39% overall). Remarkably, the mean percentage of type I fibers was significantly higher than the values for healthy adolescents of similar age. Type IIA fibers were similar, but no type IIB fibers were found; one female had only type I and type IIC fibers. In two male patients, restudied after weight gain, type IIA fibers were found to be slightly decreased, type IIB and IIC fibers were slightly increased and mean fiber area increased by 29–50%. Muscle glycogen stores were in the upper normal range and equally distributed in type I and type IIA fibers. Intramuscular lipids were mostly found in type I fibers. The activity of glycolytic and mitochondrial enzymes was lower by 50% and 20%, respectively, but capillary density was in the upper normal range. The authors propose to consider the absence of type IIB fibers a feature of the disease, arguing that only endurance exercise could increase the portion of type IIA fibers at the expense of type IIB fibers.

Lindboe et al. [50], who examined four female AN patients (mean age: 24 years; mean weight loss: 41%), all physically active, confirmed type II fiber atrophy by 75%. Type II fibers were significantly smaller than type I fibers. In contrast to Essen et al. [31], in number, type I fibers were near normal but did show atrophy by 46%. The authors attribute the differences in findings to the greater weight loss in their patients.

Similar reductions in size of type I and type II fibers, with type II fibers most severely affected, were reported by Sletteboe et al. [51] in 10 women with AN (mean age: 20.2 years; mean BMI: 15.0; mean illness duration: three years), in comparison to age- and height-matched female controls. However, fiber numbers were similar in both groups. Motor and sensory nerve conduction velocities (NCV) were not different from normal subjects; motor NCV tended to be slower in the most cachectic AN patients (BMI range: 11.4–15.2). Five AN patients with the lowest BMI had clinical signs of a mild sensory polyneuropathy. It is important to note that two older emaciated patients, diagnosed with hysterical neurosis, displayed similar type I and type II muscle fiber atrophy.

McLoughlin et al. [48] examined muscle from eight chronically and severely ill AN patients (mean age: 24 years; mean weight loss: 42%; mean BMI: 12.6). Six patients who were over-exercising admitted on questioning some muscular weakness, and five reported fatigability in the six weeks before hospital admission. The relative proportion of type I, IIA and IIB fibers was found to be normal. In diameter, type I fibers were of normal size, but severe atrophy was seen for type II fibers. Motor conduction was normal without evidence of neurogenic changes. On electron microscopy, the most prominent feature was abundant glycogen granules. A subsequent publication describing metabolic abnormalities in this group [52] found a diminished lactate response to ischemic exercise and a significant reduction of serum carnosinase activity; other abnormalities, except for lymphopenia, were not consistent.

#### 3.7.3. Metabolic Changes Related to Muscle

The relationship between thyroid status and muscle metabolism during exercise was evaluated using ^31^phosphorus magnetic resonance spectroscopy during plantar flexion exercise in eight chronic AN patients (mean age: 24.1; mean BMI: 17.3) and 13 healthy women (mean age: 21.0; mean BMI: 23.1) [53]. Serum concentrations of T_3_ and T_4_ were significantly lower in AN than in controls. Only at low intensities of steady-state exercise were small differences in the ratio of inorganic phosphate to phosphocreatine observed, suggesting that the reduction in thyroid hormones had little effect on oxidative enzyme activities in muscle. Total muscle glycogen content was 50% lower in seven AN patients, all adolescents, except for one (mean BMI: 13.6) compared to controls and returned to above normal concentrations after re-feeding. Muscle lactate levels were lower in AN patients than controls before and after refeeding [54].

Normal free and total serum carnitine levels in eight adult women with chronic AN, all of whom were vegetarians, were reported by Morton et al. [55]. Interestingly, in the very same AN patients, Mc Loughlin et al. [52] reported a reduction of carnosinase activity along with a diminished lactate response to ischemic exercise in the forearm muscle. A 50% reduction of glycolytic enzymes and a smaller reduction in mitochondrial enzymes in quadriceps muscle tissue in six AN patients were reported by Essen et al. [31].

### 3.8. Work Performance and Aerobic Work Capacity in AN

Davies et al. [56] reported significant reductions of up to 35% in maximal aerobic power (V_O2_ max) after exercise, even when calculated for lean body mass in 15 AN patients (age: 15.3; BMI: 13.8). Essen et al. [31] reported a reduction in maximal aerobic (V_O2_ max) power of 30% of control values in 10 of these patients (age: 15.3; BMI: 14.3; mean duration of illness: 1.5 years); all were physically active. In a study by Lennon et al. [57], physical work capacity on a motorized treadmill measured as metabolic equivalents was ‘significantly’ reduced in five AN patients (mean age: 17.6; mean BMI: 15.7) compared to 14 lean women (mean age: 18.8; mean BMI: 18.3). Peak work rates achieved during the progressive plantar flexion exercise test were 25% lower in eight chronic AN patients (mean age: 24.1; mean BMI: 17.3) compared with 13 controls (mean age: 21.0; mean BMI: 23.1) [53]. In 15 chronically ill and severely underweight AN patients (mean age: 29.8; mean BMI: 13.6), the workload reached during exercise on an ergometric bicycle was 49% lower than in controls, and reduced to 30% of control values (calculated as V_O2_ max). Mean urinary creatinine values were 72% of normal values. Importantly, after eight days of re-feeding, performance began to improve. After 45 days of re-feeding, with a mean 5.8 kg weight gain, work performance was similar to that in control subjects despite continued underweight [58].

### 3.9. Physical Fitness and Physical Activity in AN

Several studies [31,48,50] in AN refer to the patient sample as being physically active, i.e., walking or jogging, without reporting systematic data, perhaps because at the time of most publications, reliable activity monitors were not available.

Once spontaneous physical activity levels, conceptualized as “daily physical activity not due to exercise” [59,60] and “all physical activity behavior that emanates from an inherent drive for movement” [61], were recorded in severely underweight AN patients, no differences were found in comparison to spontaneous physical activity levels in healthy normal weight subjects [9,46,62]. For a review of these studies, see [63]. Depressive symptoms are commonly reported by AN patients. Biederman et al. [64] found that AN patients without a depressive disorder scored lower than AN patients with a depressive disorder, albeit still in the pathological range on the MMPI depression scale. The depression scores of these AN patients were in the same range as in the participants in the Minnesota experiments. Table 1 provides an overview of the findings in the reviewed studies.

## 4. Discussion

This review comparing studies in healthy humans, fasting or on restricted diets, with studies in AN patients, found similar adjustments to prolonged undernutrition in skeletal muscle morphology and muscle function in both groups. Furthermore, taking into account the reductions in strength and work capacity that characterized both groups, the reviewed studies provide no evidence for specific starvation-induced adaptations in muscle distinctive for AN which could shed light on the increased urge for movement and the preserved spontaneous activity in AN patients. The comparison does, however, highlight the unusual nature of the persistent restless activity in AN (see Table 1). The findings ought to be considered preliminary, serving as a stepping stone towards future research for investigating changes in muscle tissue in AN at the cellular and genetic level, and the pathways integrating motor, cognitive and emotional input at the motor cortex. The way in which the two groups experienced the reduction in muscle tissue and the decline in functional performance differed. Participants in the starvation experiments reported subjective physical and psychological signs (i.e., tiredness, muscle soreness, weakness, avoidance of activity), all characteristic of severe undernutrition. In contrast, studies in AN did not report subjective signs of weakness or debility; several referred to continued physical activity in patients. It is of interest that once adolescent AN patients were questioned, they did report fatigue and having no energy along with an increased urge for movement and motor restlessness, although they had not volunteered this information [10].

Overall, the experimental studies in healthy male subjects and the investigations in AN patients show that prolonged, severely calorie-deficient food intake resulting in significant body weight loss is associated with loss of muscle tissue and a decline in muscle strength and function. Adaptive changes in muscle tissue and function appear to be largely a function of the caloric deficit, with the duration of undernutrition playing a lesser role. AN patients were found to have widely varying deficient caloric intake, yet their tendency to eat some food seems to have prevented severe deterioration of muscle function until patients became dangerously cachectic.

Loss of muscle mass was significant and proportionately greater in male participants of the Minnesota experiments than in female AN patients, likely due to constitutional differences, although the overall younger age of AN patients may have contributed to preserving muscle tissue.

Concerning the ability to function on a daily basis, a moderate reduction in muscle tissue in the Carnegie experiments did not impair daily activities, albeit the men complained of leg weakness and greater effort. By contrast, at double the weight loss, the men in the Minnesota experiments experienced a significant decline both in grip strength and endurance. With work capacity and running time markedly reduced (by about 70%), many men felt debilitated by the end of the study.

In a similar way, when tested, physical performance in AN patients was found to be compromised, showing a reduction to 30–49% of the work capacity in healthy controls. The younger the patients’ age, the shorter the duration of illness, the less severe the weight loss, and the better the performance. The energy content of muscle seems to be of fundamental importance for physical performance in conjunction with metabolic factors, such as the ketogenic milieu [65], since as early as eight days after high caloric re-feeding, physical performance in AN patients was shown to improve before there were detectable changes in lean body mass [58]. AN patients typically continue to engage fully in daily activities and attend school until they are hospitalized, even when weight loss exceeds 40%.

Electrophysiological function tests of muscle strength and endurance in undernourished individuals and in AN patients showed an abnormal contractility pattern in the adductor pollicis muscle at low frequencies. This abnormal force of contraction at the lowest frequency was already recorded after five days of total fasting in healthy women and appears to be an adaptive physiological response once a critical negative metabolic balance is reached. Re-feeding completely reversed the electrophysiological changes. Muscle fatiguability was increased after two weeks of fasting, as well as in AN patients. The findings that voluntary activation of the femoris muscle was one third of normal at a mean weight loss of 42%, whereas it was comparable to healthy women at a lesser 29% weight loss, suggests that AN patients seem to be able to retain resistance capacities in leg muscles during endurance exercise, despite being considerably underweight.

Histochemical studies of the quadriceps femoris muscle in AN patients and a study in obese patients on a restricted diet concurred in finding significant muscle fiber atrophy, not only of type I fibers, albeit type II fibers were most severely affected. No signs of muscle degeneration were observed. Motor and sensory nerve conduction velocities tended to be normal, with the exception of the most cachectic AN patients, whose motor nerve conduction was slower than in controls.

Few investigators have examined muscle metabolism during undernutrition in humans. The reduced plasma carnosinase activities in patients with AN, likely due to metabolic changes in muscle and other tissues, may function to sustain normal serum carnosine levels during undernutrition [55,66]. A diminished lactate response to ischemic exercise in the forearm muscle in AN patients, which suggests the possibility of a localized defect in an aerobic glycolysis in AN, was observed only by McLoughlin et al. [52]. This finding, in conjunction with the abnormally high glycogen accumulation in muscle fibers under light microscopy [31,48], deserves further investigation. On the other hand, in another study, total muscle glycogen content was reduced by 50% in AN patients when analyzed spectrophotometrically and normalized with re-feeding [54].

## 5. Conclusions

The absence of differences in the starvation-induced adjustments in muscle between undernourished healthy individuals and AN patients can serve as an impetus for expanding the search to the cellular level, for instance to identify intramyocellular signals sensitive to nutrient availability. Since genetic factors have a significant influence on the development of AN [67], comparisons of the gene expression profile in skeletal muscle in undernourished healthy individuals and AN patients might provide new information.

We must keep in mind that the integrity of muscle, controlled by the primary motor cortex, is not the only factor determining mobility. A comparison between undernourished healthy individuals and AN patients found that perceptions of fatigue or effort are important aspects for the experience of movement. Intriguingly, two symptoms typical of AN (mental alertness and neglect of the starved body) suggest an involvement of central nervous system structures.

Therefore, a better understanding of the central nervous system neural circuitry mediating alertness and directing motor control of spontaneous activity and the sense of motor urgency might provide more information about the mechanisms underlying the persistent restless activity in AN patients.

## Figures and Tables

**Table 1:** Loss of muscle mass and adjustments in muscle morphology and muscle physiological function in underweight individuals with anorexia nervosa were found to be similar to the adaptations in muscle in response to experimental long-term starvation in healthy young adult men.

	Experimental Starvation	Anorexia Nervosa
Body weight loss	12–25%	20–40%
Muscle mass loss	20–41%	20–30%
Muscle morphology	fiber atrophy, ↓↓ type II	fiber atrophy, ↓↓ type II
Muscle physiology	↑ contractility 10 Hz	↑ contractility 10 Hz
Muscle innervation	intact	intact, unless BMI~12.5
Work capacity	↓ 20–40%	↓ 30–49%
V_O2_ max	↓	↓
Endurance	↓	↓
Increased urge to move	absent	present
Motor restlessness	rare, short-lived	continuous
Daily activity level	reduced	maintained, unless BMI~12.5
Psychological changes	present	present
Psychopathology	absent	present, multiform
